# ZnO nanoneedle/H_2_O solid-liquid heterojunction-based self-powered ultraviolet detector

**DOI:** 10.1186/1556-276X-8-415

**Published:** 2013-10-08

**Authors:** Qinghao Li, Lin Wei, Yanru Xie, Kai Zhang, Lei Liu, Dapeng Zhu, Jun Jiao, Yanxue Chen, Shishen Yan, Guolei Liu, Liangmo Mei

**Affiliations:** 1 School of Physics and State Key Laboratory of Crystal Materials, Shandong University, Jinan 250100, People’s Republic of China; 2 School of Information Science and Engineering, Shandong University, Jinan 250100, People’s Republic of China; 3 Department of Mechanical and Materials Engineering, Portland State University, P.O. Box 751, Portland OR 97207-0751, USA; 4 Department of Physics, Portland State University, P.O. Box 751, Portland OR 97207-0751, USA

**Keywords:** ZnO nanoneedle arrays, Hydrothermal method, Ultraviolet photodetector, Solid-liquid heterojunction

## Abstract

ZnO nanoneedle arrays were grown vertically on a fluorine-doped tin oxide-coated glass by hydrothermal method at a relatively low temperature. A self-powered photoelectrochemical cell-type UV detector was fabricated using the ZnO nanoneedles as the active photoanode and H_2_O as the electrolyte. This solid-liquid heterojunction offers an enlarged ZnO/water contact area and a direct pathway for electron transport simultaneously. By connecting this UV photodetector to an ammeter, the intensity of UV light can be quantified using the output short-circuit photocurrent without a power source. High photosensitivity, excellent spectral selectivity, and fast photoresponse at zero bias are observed in this UV detector. The self-powered behavior can be well explained by the formation of a space charge layer near the interface of the solid-liquid heterojunction, which results in a built-in potential and makes the solid-liquid heterojunction work in photovoltaic mode.

## Background

Ultraviolet (UV) detectors play an essential role in a wide range of civil and military applications including UV astronomy, environmental monitoring, flame sensing, secure space-to-space communications, and chemical/biological analysis [1-3]. As a wide bandgap material, ZnO has emerged as one of the most promising materials for UV detectors due to its exceptional photosensitivity and high radiation hardness [4-6]. ZnO has a direct wide bandgap of 3.37 eV, eliminating the need for costly filters to achieve visible-blind operation as that in traditional photomultipliers and silicon photodetectors. Its bandgap can be tuned in a wide range simply by doping with a small mole fraction of Al, Mg, or Cd, which enables ZnO to be used in different detection ranges. In the past, most ZnO-based photodetectors were fabricated in planar type based on ZnO thin films grown by sputtering, pulsed laser deposition, or molecular beam epitaxy. Different kinds of UV detectors based on ZnO have been investigated with metal-semiconductor-metal [7-10], p-i-n [4,11,12], p-n junction [5,13,14], or Schottky barrier-type [15-17] structures. However, factors such as high cost, difficulty of integrating with Si substrate, and complicated fabrication process have drawn back the potential application of planar-type ZnO photodetectors.

Recently, there is a growing interest in UV detectors based on one-dimensional (1D) nanostructures of ZnO like nanowires [18-20] or nanobelts [21] due to the highly susceptible photoelectric properties by means of electron-hole generation or recombination under UV illumination. ZnO nanowire-based UV sensors exhibit a high on/off ratio between photoresponse current and dark current because of the large surface-to-volume ratio and the high crystal quality. Additionally, characteristics such as fast response and recovery time, visible light blindness, and potential for flexible electronics [22,23] further contribute to 1D UV detectors' competence. However, the very low photoresponse current due to the small size of individual nanowires is an essential hindrance to single ZnO nanowire-based UV detectors [18,20,24]. Efficient routes like integrating multiple nanomaterials or assembling nanoarrays often lead to a complicated, time-consuming, and uneconomic device fabrication process [24-26]. On the other hand, these photodetectors typically require an external bias as the driving force to prevent the recombination of photogenerated electron-hole pairs. For large-area two-dimensional arrays that contain huge amounts of small UV sensors, large-scale use of batteries as a power source will lead to environmental pollution [27-29].

In this letter, we introduce a self-powered UV detector based on a ZnO nanoneedle/water solid-liquid heterojunction structure. ZnO nanoneedle arrays were grown on a fluorine-doped tin oxide (FTO)-coated glass substrate by spin coating and subsequent hydrothermal method without any costly epitaxial process. X-ray diffraction (XRD) and scanning electron microscope (SEM) results proved a high-quality, vertically aligned ZnO nanoneedle array structure. A self-powered photoelectrochemical cell-type UV detector was assembled using the ZnO nanoneedles as the active photoanode and H_2_O as the electrolyte, which has almost the same structure as that of a conventional dye-sensitized solar cell but without dye adsorption. The solid-liquid heterojunction owes an inherent built-in potential across the interface which behaves in a Schottky barrier manner. The built-in potential acts as the driving force to separate the electron-hole pairs from recombination and generate photocurrent [28-30]. Hence, this ZnO/water heterojunction-based UV detector operates in photovoltaic mode, eliminating the need for external electric bias, which demonstrates a great potential in realizing self-powered UV detection and a self-driven integrated nanopower-nanodevice system [31].

## Methods

### Growth of ZnO nanoneedle arrays by hydrothermal process

ZnO nanoneedle arrays were grown using solution deposition method on FTO glass covered with a ZnO seed layer. Zinc acetate dehydrate was dissolved in the mixed solution of ethanolamine and 2-methoxyethanol to yield a homogeneous and stable colloid solution, which served as the seed solution. The ZnO seed layer was formed by spin coating the colloid solution at 3,000 rpm followed by annealing in a furnace at 400°C for 1 h. The following hydrothermal growth was carried out at 90°C for 6 h in a Teflon bottle by placing the seeded substrates vertically in aqueous growth solutions, which contain 20 mM zinc nitrate, 20 mM hexamethylenetetramine, and 125 mM 1,3-diaminopropane. Then the FTO glass with ZnO nanoneedle arrays was rinsed with deionized water thoroughly and annealed at 500°C for 1 h to remove any residual organics and to improve the crystalline structure.

### Assembly of the solid-liquid heterojunction-based UV detector

**Figure 1:**
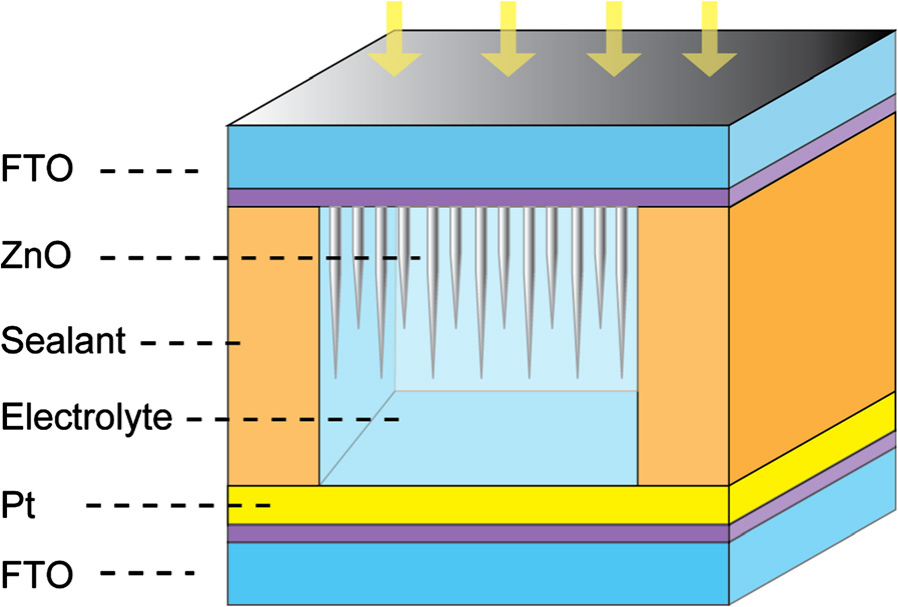
Schematic device structure of the ZnO nanoneedle array/water solid-liquid heterojunction-based ultraviolet photodetector.

The solid-liquid heterojunction-based UV detector was assembled in the same structure as that of a dye-sensitized solar cell, except that no dye molecules were adsorbed and the electrolyte used in this case was deionized water, as discussed in our previous work [32]. Figure 1 shows the schematic structure of the nanocrystalline ZnO/H_2_O solid-liquid heterojunction-based UV detector. For device manipulation, FTO glass with vertically aligned ZnO nanoneedle arrays was used as the active electrode. A 20-nm-thick Pt film deposited on FTO glass by magnetron sputtering formed the counter electrode. Afterwards, the work electrode (ZnO/FTO) and the counter electrode (Pt/FTO) were adhered together face to face with a 60-μm-thick sealing material (SX-1170-60, Solaronix SA, Aubonne, Switzerland). Finally, deionized water was injected into the space between the top and counter electrode. A ZnO/H_2_O solid-liquid heterojunction-based UV detector was fabricated with an active area for UV irradiation of about 0.196 cm^2^.

### Characterization of ZnO nanoneedle arrays and the UV photodetector

The crystal structure of the ZnO nanoneedle arrays was analyzed by XRD (XD-3, PG Instruments Ltd., Beijing, China) with Cu Kα line radiation (*λ* = 0.15406 nm). The surface morphology was characterized using a scanning electron microscope (Hitachi S-4800, Hitachi, Ltd., Chiyoda, Tokyo, Japan). The optical transmittance was measured using a UV-visible dual-beam spectrophotometer (TU-1900, PG Instruments, Ltd., Beijing, China). The photoresponse characteristics of the UV detector under illumination were recorded with a programmable voltage-current sourcemeter (2400, Keithley Instruments Inc., Cleveland, OH, USA). A 500-W xenon lamp (7ILX500, 7Star Optical Instruments Co., Beijing, China) equipped with a monochromator (7ISW30, 7Star Optical Instruments Co.) was used as the light source. For the photoresponse switching behavior measurement, photocurrent was measured by an electrochemical workstation (RST5200, Zhengzhou Shirusi Instrument Technology Co. Ltd, Zhengzhou, China).

## Results and discussion

**Figure 2:**
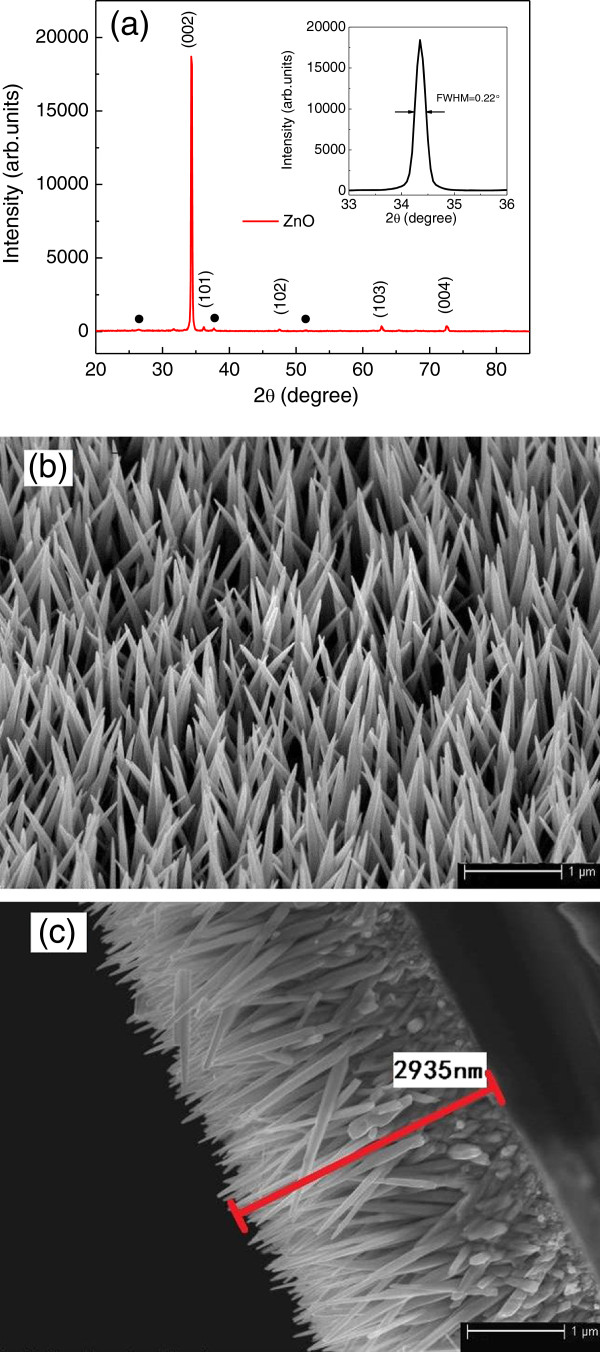
**XRD pattern and SEM images of ZnO nanoneedle arrays. (a)** X-ray diffraction pattern of the ZnO nanoneedle arrays grown on FTO glass; the inset shows the magnified image of a wurtzite ZnO (002) peak with a FWHM of 0.22°. **(b)** Tilted-view FESEM image (40° tilted) of the ZnO nanoneedle arrays grown on FTO glass by hydrothermal method. **(c)** Cross-sectional-view FESEM image of the ZnO nanoneedle arrays.

Figure 2a shows the typical XRD pattern of ZnO nanoneedle arrays grown on FTO substrate. All of the diffraction peaks can be indexed within experimental error as a hexagonal ZnO phase (wurtzite structure) from the standard card (JCPDS 76-0704). No characteristic peaks from impurities such as Zn(OH)_2_ are detected. Compared to powdered ZnO XRD patterns, the (002) diffraction peak was significantly enhanced, which indicates that the ZnO nanoneedles are highly oriented along the *c*-axis direction with the growth axis perpendicular to the substrate surface. The full width at half maximum (FWHM) of ZnO (002) is 0.22° as shown in the inset of Figure 2a, demonstrating the good crystallinity of the ZnO nanoneedles. The tilted-view and cross-sectional SEM images of as-grown ZnO nanoneedle arrays are shown in Figure 2b,c. The images at different locations and viewing angles reveal that the entire surface of the FTO-coated glass substrate is uniformly covered with ordered ZnO nanoneedles. The SEM image clearly shows that ZnO nanoneedles with sharp tips are grown vertically on the FTO substrate. Further analysis indicates that the average length of the nanoneedles is about 2 to 3 μm and the diameters are 80 to 100 nm at the base, which can be controlled by the growth time and DAP concentration in the aqueous growth solution.

**Figure 3:**
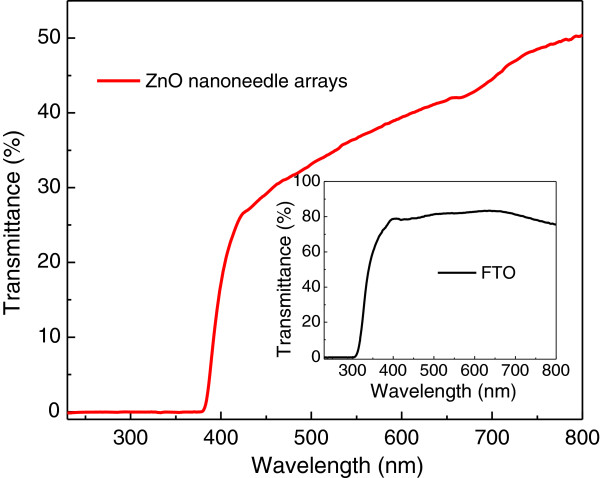
The UV-visible transmittance spectra of the ZnO nanoneedle array and a typical FTO glass substrate (inset).

As is shown in Figure 3, the optical property of the ZnO nanoneedle arrays was characterized by the UV-visible transmittance spectrum in the range of 220 to 800 nm. In the visible light region, ZnO shows low transmittance (30% to 50%), which comes from the strong light scattering effect of the nanoneedle array structure. An obvious sharp absorption edge appears at about 385 nm, which can be attributed to the bandgap of wurtzite ZnO nanoneedle arrays. Not much difference can be found in the absorption edge of the nanocrystalline ZnO as compared with that of bulk ZnO in this case, as the size of the ZnO nanoneedle is well above the ZnO Bohr exciton diameter. The inset of Figure 3 shows the transmittance spectrum of a typical FTO substrate, with an average transmittance of 80% within the visible light region and a sharp absorption edge at about 310 nm. Taking both the absorption spectra of ZnO and FTO glass into consideration, we can achieve the conclusion that light with a wavelength of 310 to 385 nm can be well absorbed by ZnO nanoneedle arrays and contribute to the photoresponse, which is further confirmed by the following photoresponsivity spectrum.

**Figure 4:**
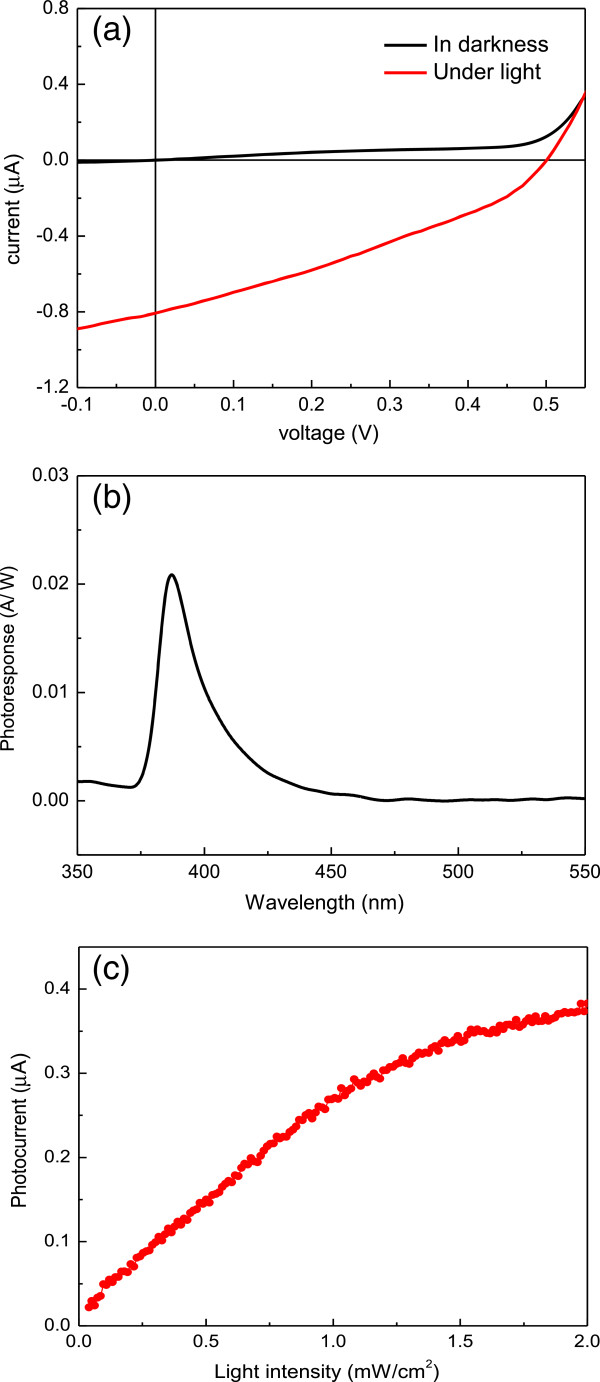
**Photoresponsivity of the ZnO nanoneedle array/water UV detector. (a)** Typical *I*-*V* characteristics of the ZnO nanoneedle array/water UV photodetector in darkness and under the illumination of 1.25 mW/cm^2^ of UV light (*λ* = 365 nm). **(b)** Spectral responsivity characteristic of the UV detector under 0-V bias. **(c)** Photoresponse current versus UV illumination intensity under 0-V bias and 365-nm UV light irradiation.

Typical current-voltage (*I*-*V*) characteristics of the UV detector in darkness and under UV illumination are shown in Figure 4a. Under the illumination of 1.25 mW/cm^2^ of UV light (*λ* = 365 nm), this solid-liquid heterojunction-based UV detector shows an excellent photovoltaic performance, yielding a short-circuit current (*I*_sc_) of 0.8 μA and an open-circuit voltage (*V*_oc_) of 0.5 V. This inherent built-in potential arises from the SB-like ZnO-water interface, acts as a driving force to separate the photogenerated electron-hole pairs, and produces the photocurrent. Therefore, this device can operate at photovoltaic mode without any external bias. Figure 4b shows the spectral photoresponsivity of the ZnO nanoneedle array/water heterojunction-based UV detector at 0-V bias. The incident light wavelength ranges from 350 to 550 nm. A strong peak appears at 385 nm, corresponding to the bandgap of wurtzite ZnO. The maximum responsivity located at around 385 nm is about 0.022 A/W cm^2^, which is suitable for UV-A range (320 to 400 nm) application. Note that the full width at half maximum of the photoresponse is about 18.5 nm (0.15 eV) as shown in Figure 4b, which demonstrates excellent spectral wavelength selectivity in the UV-A range. The photoresponsivity decreases rapidly to nearly zero as the wavelength is longer than 450 nm because of the low absorption for photons with energies smaller than the bandgap. The responsivity also drops fast on the short-wavelength side because of the strong electron-hole recombination effect. As illustrated in Figure 2c, the ZnO nanoneedle array has a dense, compact layer at the base (closest to FTO). The absorption coefficient of ZnO at a wavelength shorter than 375 nm is very high. When illuminated through the FTO glass, the majority of photons will be absorbed by this ZnO layer close to the FTO. This absorption occurs well away from the junction. Due to the high electron-hole recombination rate in this layer, only carriers excited near the junction region contribute to the photocurrent in the photodetector. Therefore, UV light below 375 nm only creates a poor photocurrent response. The photocurrent under different incident light intensities was also measured. The measurement of this self-powered UV detector was carried out at 0-V bias and under 365-nm UV light irradiation. As shown in Figure 4c, under weak UV light intensity, the photocurrents are almost linearly increased with an increasing incident UV light intensity. A gradual saturation of the photocurrent was observed under higher UV irradiances. One possible reason for this saturation is the poor hole transport ability of water.

**Figure 5:**
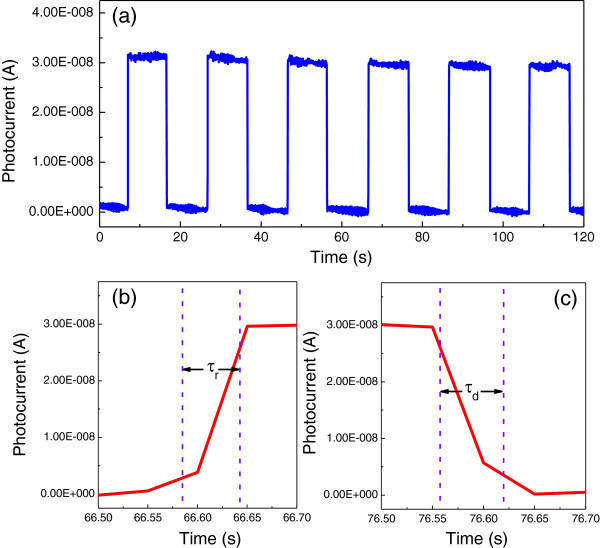
**The real-time photocurrent response of the ZnO nanoneedle array/water UV detector. (a)** Photocurrent response under on/off UV light radiation with the illumination wavelength of 385 nm. Enlarged **(b)** rising edge and **(c)** decaying edge of the photocurrent response.

The real-time photocurrent response of the self-powered UV detector at 0-V bias is shown in Figure 5 under an incident UV light with a wavelength of 385 nm, corresponding to the bandgap of ZnO nanoneedle arrays. The incident radiation is switched with an on/off interval of 10 s. Six repeated cycles are displayed in Figure 5a, in which the photocurrent is observed to be consistent and repeatable with no degenerate effect found during the detection process. From the magnified rising and decaying edges of photocurrent shown in Figure 5b,c, respectively, a fast photoresponse can be seen clearly. The rising time (defined as the time to increase from 10% to 90% of the maximum photocurrent) and the decaying time (defined as the time to recover from 90% to 10% of the maximum photocurrent) are both approximately 0.1 s, indicating rapid photoresponse characteristics.

**Figure 6:**
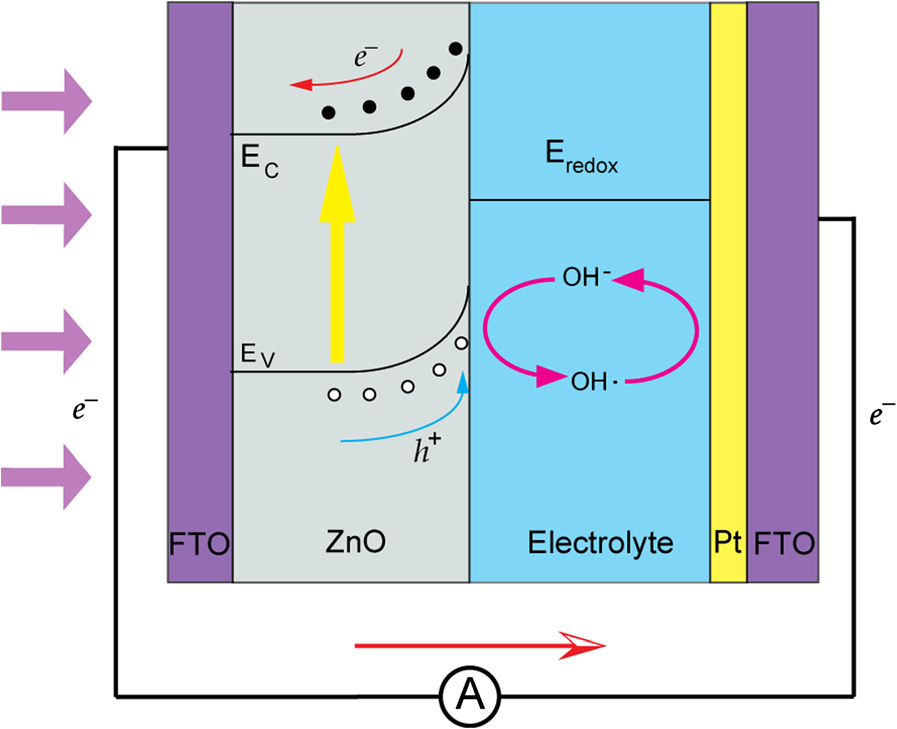
Energy band diagram and working principle for the UV photodetector under 0-V bias and illumination.

In order to clearly clarify the working principle of this self-powered UV detector, a simple energy band diagram is schematically shown in Figure 6. Since the Fermi level of the n-type semiconductor (ZnO) is higher than the redox potential of the aqueous electrolyte (deionized water), when a semiconductor is placed in contact with an electrolyte, electric current initially flows across the junction until electric equilibrium is reached [28-30]. In this case, electrons will transfer from the semiconductor (ZnO) into the electrolyte (deionized water), which will produce a region on each side of the heterojunction where the charge distribution differs from the bulk material, known as the space charge layer. Electron depletion from solid into the solution results in a positive excess charge by immobile ionized donor states. Hence, an electric potential difference across the solid-liquid interface is set up, which works in a Schottky barrier mode, as is reflected by the upward bending of the bandgaps of the n-type semiconductor.

When incident light travels through FTO glass and reaches the active layer of ZnO nanoneedle arrays, photons with energy exceeding that of the ZnO bandgap will be absorbed and electron-hole pairs will be generated thereafter. The built-in potential across the interface works as the driving force to separate the electron-hole pairs. Negative charge moves along the ZnO nanoneedle and gets collected by the FTO electrode and poured into the external circuit easily since the work function of FTO matches with the conduction band of ZnO. The positive holes are driven to the surface and got captured by the reduced form of the redox molecule (h^+^ + OH^-^ → OH·). Fast removal of holes can be expected across the heterojunction due to the large surface area. The oxidized form of the redox molecule is reduced back to the reduced form OH^-^ at the counter electrode (Pt/FTO) by the electrons that re-entered into the UV detector from the external circuit (e^-^ + OH· → OH^-^). The circuit was completed in this manner, demonstrating a self-powered UV detection property.

Overall, the ZnO nanoneedle array/water solid-liquid heterojunction is one type of regenerative UV detector. Considering the tunability of the absorption edge of ZnO by simply changing the concentration of the doping element like Al [33,34] or Mg [35,36] and excellent spectral selectivity of this system, we suggest that the spectral response should be tailored by elemental doping [37] in a relatively wide range, which presents a promising versatile potential. In addition, the photoresponsivity and time performance of the solid-liquid heterojunction can also be improved by seeking for the optimized electrolyte solution. The simple fabrication technique, low cost, and environmental friendliness (nontoxic composition) further add to the solid-liquid UV detector's commercial application.

## Conclusion

In conclusion, *c*-axis-preferred ZnO nanoneedle arrays have been successfully prepared on a transparent conductive FTO substrate via a simple hydrothermal method. A new type of self-powered UV detector based on a ZnO nanoneedle array/water solid-liquid heterojunction structure is fabricated, which exhibits a prominent performance for UV light detection. The photocurrent responds rapidly with UV light on-off switching irradiation under ambient environment. The mechanism of the device is suggested to be associated with the inherent built-in potential across the solid-liquid interface which works in a Schottky barrier manner that separates the electron-hole pairs generated under UV irradiation. The large relative surface and high crystal quality further promote the photoresponse. This new type of self-powered solid-liquid heterojunction-based UV detector can be a particularly suitable candidate for practical applications for its high photosensitivity; fast response; excellent spectral selectivity; uncomplicated, low-cost fabrication process; and environment-friendly feature.

## Competing interests

The authors declare that they have no competing interests.

## Authors’ contributions

The work presented here was performed in collaboration of all authors. QL carried out the measurements of the TNA/water UV detector and drafted the manuscript. LW grew the ZnO nanoneedle array. YX carried out the XRD and SEM characterizations. KZ conducted the transmittance spectra measurements. LL and DZ deposited the Pt film and helped fabricate the device. YC supervised the work and finalized the manuscript. GL and SY analyzed the results and participated in the revision of the manuscript. LM and JJ proofread the manuscript and corrected the English. All authors read and approved the final manuscript.
